# COVID-19 Impact on Community-Based Participatory Randomized Controlled Trials—Lessons From the Oral Health Disparities in Children Consortium

**DOI:** 10.3389/fdmed.2021.671911

**Published:** 2021-07-09

**Authors:** Francisco J. Ramos-Gomez, Molly A. Martin, Suchitra S. Nelson, Belinda Borrelli, Michelle M. Henshaw, Shelley Curtan, Helen E. Lindau, Nicolle Rueras, Anna S. Sandoval, Stuart A. Gansky

**Affiliations:** 1 Department of Pediatric Dentistry, University of California, Los Angeles, Los Angeles, CA, United States,; 2 Center to Address Disparities in Children’s Oral Health (CAN DO), University of California, San Francisco, San Francisco, CA, United States,; 3 Institute for Health Research and Policy, University of Illinois Chicago, Chicago, IL, United States,; 4 Department of Community Dentistry, Case Western Reserve University, Cleveland, OH, United States,; 5 Center for Behavioral Sciences Research, Henry M. Goldman School of Dental Medicine, Boston University, Boston, MA, United States,; 6 Global and Population Health, Henry M. Goldman School of Dental Medicine, Boston University, Boston, MA, United States,; 7 School of Dentistry, University of California, San Francisco, San Francisco, CA, United States

**Keywords:** COVID-19, pandemic (COVID-19), clinical trials, community-based participatory research, dentistry, behavioral science, health disparities, health inequities

## Abstract

The COVID-19 pandemic has had a major impact on nearly every sector of science and industry worldwide, including a significant disruption to clinical trials and dentistry. From the beginning of the pandemic, dental care was considered high risk for viral transmission due to frequent aerosol-generating procedures. This resulted in special challenges for dental providers, oral health care workers, patients, and oral health researchers. By describing the effect that the COVID-19 pandemic had on four community-based randomized clinical trials in the Oral Health Disparities in Children (OHDC) Consortium, we highlight major challenges so researchers can anticipate impacts from any future disruptions.

## INTRODUCTION

In the beginning of the COVID-19 pandemic, roughly 80% of non–COVID-related trials were interrupted or stopped (1). The nature of COVID-19, a highly contagious respiratory virus spread *via* respiratory droplets, requires handwashing, masking, and social (physical) distancing—even with quarantines—to prevent viral spread. These measures create many complications for clinical trials with in-person visits for intervention delivery and/or data collection (2, 3). From the beginning of the pandemic, dental care was considered high risk for viral transmission due to aerosol-generating procedures that are frequently used. Dental providers and oral health care workers were at high risk for both being infected by, and spreading, COVID-19. This risk carried over into dental research.

The Oral Health Disparities in Children (OHDC) Consortium is composed of four separate trials focused on the oral health of US children. All trials receive support from a single Coordinating Center (CC) that provides data acquisition and quality assurance services. The OHDC Consortium reflects the National Institute for Dental and Craniofacial Research’s (NIDCR) efforts to support research using community engagement to answer scientific questions. Each study addresses various health determinants and risk factors at multiple influence levels, such as individual, family, community, organization/institution, health services, and public policy.

The four OHDC Consortium trials include the BEhavioral EConomics for Oral health iNnovation (BEECON) trial (ClinicalTrials.gov registration number NCT03576326), the Coordinated Oral Health Promotion Chicago (CO-OP) trial (NCT03397589), the Interactive Short Messages to Initiate Lasting Education (iSmile) trial (NCT03294590) and the Pediatric Providers Against Cavities in Children’s Teeth (PACT) trial (NCT03385629). The BEECON trial tests financial incentives for families to improve oral health behaviors that could prevent early childhood caries. BEECON is conducted jointly by University of California San Francisco (UCSF) and University of California Los Angeles (UCLA), with participants in Los Angeles County, CA (4). The CO-OP trial tests a family-focused community health worker program. CO-OP Chicago is led by the University of Illinois at Chicago (UIC) with participants from Cook County, IL (5). The iSmile trial tests an interactive, automated, and customized parent-targeted text message program to improve oral health among children attending urban pediatric clinics. iSmile is led by Boston University, Henry M. Goldman School of Dental Medicine (BU-GSDM) with participants in Boston, MA (6). The PACT trial tests bundled practice- and provider-level interventions in primary care that include oral health education, referral to dental care, and integrating oral health assessment into electronic health records. PACT is conducted by Case Western Reserve University (CWRU) in Cleveland, OH (7).

While all four OHDC Consortium community-based prevention trials had already completed enrollment before COVID-19 began to spread widely in the USA, the pandemic greatly disrupted in-person follow-up visits that were integral to collecting primary outcome variables (e.g., oral health examinations to assess dental caries, dental plaque assessments). This paper aims to discuss the impact that the COVID-19 pandemic had on conducting the OHDC Consortium trials specifically, and on community-based participatory research, in general. Moreover, this paper provides recommendations for modifying community-based research during future unforeseen disruptions.

## MATERIALS AND METHODS

### Data Collection and Analysis

In March, the states in which the studies were conducted (California, Illinois, Ohio, and Massachusetts) implemented stay-at-home orders which required the OHDC Consortium trials to pause in-person visits. All projects had to cease in-person research in March due to the shut-down, but each project had very different timelines for restart given the various approvals required at universities and at external sites. From February 2020 through January 2021, the standing monthly OHDC Consortium Steering Committee (SC) meeting of Principal Investigators (PIs) and lead staff from each trial, the CC, and NIDCR project scientists became an important virtual venue to discuss the impacts of COVID-19 on trial operations and shared strategies for how to address pandemic-caused interruptions. Based on information documented during these discussions, COVID-19 specific barriers and strategies were compiled to describe the main issues the OHDC Consortium SC identified. Staff from the CC distributed the notes that were compiled and each study team contributed to the list of barriers and strategies. Then the PIs reviewed these qualitative data and consolidated the information into four domains as shown in Table 1: timeline adjustments; data collection, retention, and analysis; logistical challenges; scientific challenges.

## RESULTS

### Domain 1: Timeline Adjustments (Table 2)

#### BEECON

All in-person clinical components were halted on March 16, 2020, when UCLA shut down research, following stay-at-home orders in Los Angeles County and remained halted until October 1, 2020, when clinic visits were approved to resume. BEECON text messages regarding the incentive intervention continued without impact during COVID-19. Visits occurred daily from October 1 to October 19 and then were approved to resume twice per week starting November 6. Subsequent holiday related surges in Los Angeles County caused clinic visits to be halted again from January 6, 2021 to February 25, 2021.

The reviewing IRB (UCSF) posted guidance about COVID-19 related study modifications not needing pre-approval in minimal risk studies, which included changes from in-person to remote data collection. No modification was required until the annual Continuing Review since the changes did not increase participant risk. Therefore, where possible questionnaires that could be administered over the telephone were used so some data collection could continue, only pausing clinical visit portions of data collection (dental caries screening, plaque disclosing, plaque photograph, and fluoride varnish application). Study staff mailed participants supplies (toothbrush heads, tooth paste pumps, brushing calendars, and written materials). In January 2021, the trial submitted an IRB modification to extend the 12-month data collection visit window for an extra 90 days due to clinic closures caused by the winter COVID-19 surge in Los Angeles County.

#### CO-OP

The CO-OP timeline was delayed a total of 90 days. When UIC shut down on March 16, 2020, CO-OP Chicago had completed all intervention delivery and had follow-up data collection pending on only 38 of enrolled 420 participants. On April 19, 2020, CO-OP Chicago received IRB approval extending data collection visit windows another 90 days. Starting April 21, 2020, the study team resumed data collection *via* phone, reaching 21 participants. One participant did not want any further data collection beyond the phone visit, 20 agreed to attempt the extended window period. When home visits clearly would not be in the best interests of staff and participants for the foreseeable future, CO-OP Chicago obtained IRB approval (on July 6, 2020) for families to complete the objective data collection themselves with trial staff supporting them by applying disclosing solution and using their smartphones to take photographs. The final data collection window then closed and all data collection subsequently ended August 20, 2020.

#### iSmile

In-person study visits were suspended on March 12, 2020, when the University halted all in-person research. The iSmile intervention is delivered electronically *via* text messages, so there was no disruption in intervention delivery. Because the original protocol included participant completion of online questionnaires remotely, this portion of the study was not affected by the shut-down. The primary outcome variable, however, is collected during in-person visits at four pediatric clinics, which each had a different timeline for resumption of research activities. In all cases, clinics resumed patient care but indicated that they needed to delay resumption of research. Before resuming research activities, iSmile staff had to obtain approval from both the University and from the medical campus of the University because it was clinical research conducted off-campus. The approval process required submission of different forms that detailed the precautions for participants and staff, including personal protective equipment (PPE), social distancing, travel, and scheduling.

Once approved, the team met with leadership from each clinic *via* Zoom to discuss restart guidelines and timelines. iSmile received approval to resume research at its first site on September 10, 2020, and its second on October 1, 2020. Due to the COVID-19 surge that began in late 2020, the January 1, 2021 start date to resume research at the third site was further delayed. During the pandemic, the final site underwent a merger which required new approvals be attained and agreements drafted, reviewed, and executed. Research restart is expected March 2021 in the final two sites. Importantly, different protocols have been required for each clinic regarding staff COVID-19 testing and vaccination, participant scheduling, participant COVID-19 screening, participant arrival, use of waiting rooms, and inter-participant intervals. Study visit windows were extended because of the lengthy suspension of in-person research activities.

#### PACT

The PACT study conducts in-person visits at the time children are seen for their well-child visits (WCVs). At the WCVs, the pediatricians/nurse practitioners deliver the intervention, and study staff complete questionnaires with parents and conduct the clinical dental screening examination for the child. Between March 20, 2020 and May 25, 2020, all in-person follow-up WCVs were paused in accordance with the CWRU, University Hospitals, and Ohio guidelines. When the shutdown began, 666 (65%) and 349 (6%) of 1,024 participants were seen for their subsequent Well Child Visit (WCV) 2 and 3. During this period, some participants were seen at WCVs that the study staff missed due to COVID-19 restrictions. However, questionnaire data were still collected by mailing paper forms for parents/caregivers to complete and return. US Postal Service delays during the pandemic were slightly more frequent for participants receiving forms and the study receiving completed forms from participants; however, this had a minor impact on data collection. In-person visits resumed on May 26, 2020. Again, due to staff being quarantined from October 5 to 19, 2020 all in-person WCVs were paused. The same protocol was followed to obtain questionnaire data for visits that were missed during this period. In-person visits resumed on October 20, 2020. Visit windows for the PACT study are ±3 months of the follow-up WCVs. In addition, the PACT protocol had specified from the beginning that visits may occur out-of-window and that the duration between visits will be accounted for in the analysis. Therefore, the necessity to extend the windows was not an issue with the PACT study.

### Domain 2: Data Collection, Retention, and Analysis

#### Data Collection Innovations

The BEECON and CO-OP trials before COVID-19 had been collecting images of children’s teeth after applying a disclosing solution to assess plaque. Research staff conducted this process in homes (CO-OP) and at the clinic (BEECON). Because in-home data collection could not resume before study follow-up would end, the CO-OP study delivered plaque disclosing solution and asked the parents to apply the solution and collect the images. Of the 20 families eligible, 6 completed the process and submitted adequate images for plaque assessment. The BEECON staff created infographics, educational videos, and other resources that would guide participants how to apply the disclosing solution and take plaque photographs, but did not ultimately use them due to the return to the clinic for in-person visits.

#### Retention Improvements

Before COVID-19, the BEECON trial relied on participants to come to clinic for data collection. With the COVID-19 modifications, the BEECON study saw a boost in retention as data collection was able to be completed telephonically. The iSmile trial had an increase in the number of 12- and 24-month self-report surveys that were completed during the stay-at-home order. Study staff in both trials had more time to devote to retention activities because in-person oral health assessments were suspended.

Furthermore, participants were more easily reached *via* text message and phone during the stay-at-home orders. In the PACT trial, retention rates were slightly decreased with some participants not returning questionnaires to the study team even with multiple calls to the participants. Also, even with WCVs resuming in June 2020, some parents were hesitant to schedule their children’s WCVs or canceled visits. Consequently, this extended study windows for follow-up visits.

#### Missing Data

Before the COVID-19 pandemic, these trials expected that data would be missing at random (MAR) and the failure to complete the oral health examination would be expected to depend on participant characteristics. Due to the COVID-19 pandemic, the data would be missing completely at random (MCAR) because the clinics were closed and participants could not attend their visits; therefore, missing data are not dependent upon participant characteristics. During analysis, models will have to account for both types of missing data. These assumptions would then drive the choice of analytic model. For example, these trials cannot use inverse probability weighting to account for missing data because the weights are based on the propensity for missing data, and that changes occurred during the trial. Multiple imputation may be a better approach for analysis for these trials which is a departure from the original plan to do Inverse Probability Weighting (IPW).

#### Analysis Modifications

The BEECON trial modified data collection to allow for fewer data collection visits if more than one planned in-person data collection was missed during COVID-19. Final analyses for the primary endpoint of toothbrushing behavior will use the planned visit times. Final analyses of the secondary endpoints of caries screening and plaque from photo of disclosed F incisor teeth will use actual dates to account for the delays due to COVID-19. Final analyses for the CO-OP trial will be conducted both with and without the 21 participants whose data were collected after COVID-19 started due to concerns their data would not adequately represent their normal experiences. Due to the extended window for data collection, the iSmile trial will need to add a time-to-event analysis. For example, because a large number of participants could not complete their 24-month oral health examination within window, and because this is the primary outcome variable, iSmile expanded the window end date and will control for time since baseline in analyses. Time of the oral examination (months from baseline) will be included as a predictor since caries increases with age; for a logistic regression analysis predicting any caries, this adjusts for the chance of any caries increasing with oral examinations later than planned. For the primary analysis at 24 months, this is just another predictor. In longitudinal analyses assessing oral examination data from baseline, to 12–24 months, this could be a time-dependent covariate, reflecting the timing of the 12-month examination and the timing of the 24-month examination. The PACT study will use Medicaid data for its primary endpoint which will be available for almost all participants regardless of WCV attendance. The PACT study will have missing data for the secondary endpoints using questionnaire data. PACT will use actual time between baseline and follow-up visit to perform longitudinal analysis.

### Domain 3: Logistical Challenges

The COVID-19 pandemic presented logistical challenges primarily related to personal protective equipment (PPE), participant compensation, and staffing that required changes to study protocols.

#### PPE and Risk of Aerosolization

Before COVID-19, BEECON trial PPE requirements for dental providers included surgical masks, safety glasses, disposable gowns, and gloves, while research assistants were not required to wear any PPE. To reduce aerosolization risk after COVID-19, the in-person data collection protocol was revised. Dental providers were instructed not to dip the toothbrush in water before removing the disclosing solution from the participating child’s teeth to reduce possible aerosols. Dental providers instead used a dry toothbrush or gauze to remove the disclosing solution from the child’s teeth. When in-person data collection resumed in October 2020, PPE requirements included disposable gowns, surgical masks, face shields, safety glasses, and gloves for both the dental providers and research assistants. Dental providers also wore surgical bonnets and had the option to wear N95 masks, which were not required because BEECON dental screenings are not aerosol-generating procedures. There were no issues obtaining necessary PPE for the BEECON Study.

The iSmile data collection protocol includes dry brushing the teeth before the oral health assessment; plus, the participants’ young age frequently resulted in crying during the examination; both of these produce aerosols. A decision was made not to change the clinical examination protocol. Therefore, after COVID-19, the PPE required for the clinical examiners changed from surgical masks, disposable gowns, safety glasses, and gloves to also include an N95 mask covered with a surgical mask, face shield, surgical bonnet, and surgical booties.

The research assistants use the same PPE as the clinical examiners since maintaining a 6-foot distance from the participant was challenging when recording data. Parents with children also had to wear a surgical mask.

PPE, especially N95 masks, were not available when research was restarted due to limited global supply. This was one of the limiting factors in approval to resume in-person research visits.

PACT trial data collection was conducted in primary care clinics, but included a dental examination with tooth brushing with a potential risk of aerosolization. Shortly after returning to visits, primary care physicians did not have N95 masks available and therefore study staff had more PPE concerns. To avoid conflict, the study team changed the protocol to stop tooth brushing children and to use gauze to wipe teeth before examination, thereby reducing aerosolization risk. With this change, the hygienists were comfortable using a surgical mask, face shield, gowns, gloves to conduct the brief dental examination. Primary care clinics later changed their requirements with all study hygienists required to wear a N95 mask while examining the child.

The CO-OP trial completed data collection; therefore, it did not have to secure any PPE.

#### Participant Compensation Reimbursement

Before COVID-19, the BEECON trial provided $30 in grocery store gift cards to participants for attending clinic visits. When BEECON moved to telephonic data collection in March 2020, participants were offered electronic gift cards for payment immediately or participants could receive their grocery store gift cards at a later date when the clinic reopened. Reimbursement did not depend on completing plaque images to be collected at clinic visits later. The delay for clinic reopening became longer than expected, resulting in the BEECON team coordinating contactless delivery of grocery store gift cards to participants who had chosen that option.

In the CO-OP trial, reimbursement changed from cash at data collections to a check for the same amount that was sent *via* mail. Participants were reimbursed immediately after the telephone data collection and reimbursement was not dependent on completing plaque images attempted later.

While the total amount of participant compensation remained constant in the iSmile trial, timing of the compensation changed during COVID-19. Before COVID-19, participants received a $60 gift card after completing both the online questionnaire and the child’s oral health assessment. This was adjusted to give a $30 electronic gift card upon completing the online questionnaire and an additional $30 gift card when in-person oral health assessments were completed later after resuming in-person research activities. This change was initiated to motivate remote questionnaire completion while waiting for permission to restart the study in the clinic.

In the PACT trial, participants received cash at WCVs and after completing follow-up questionnaires. The only change made was that the incentive was mailed to the participants instead of distributing in person. After participants signed a mailed receipt, the money was mailed to them. The PACT trial did not have any instances of people reporting not receiving the incentive after mailing.

#### Staffing Changes

The BEECON trial staff did not change its hours or staffing numbers, although all moved remotely once the stay-at-home was declared. Because in-person visits could not happen, project staff and providers usually responsible for those activities assisted the project manager with designing a drive-thru dental clinic option that was ultimately not implemented.

When the stay-at-home order was initiated in March 2020, some CO-OP staff had to convert computer equipment to allow for effective working at home. This included hooking up computers to TV monitors and purchasing home office supplies like chairs. Before COVID-19, the plan had been for the intervention and research assistant staff positions to end in Spring 2020 as intervention and data collection ended. During the initial months of the pandemic, UIC put a hold on all staffing changes. The extended data window also made it necessary to retain research staff longer. Therefore, employment for five full-time staff continued for many months despite almost no work for them, impacting the study budget.

iSmile had no reduction in its research staff. All study staff and investigators moved to working remotely, in accordance with stay-at-home orders. Research assistants continued working on retention activities and ensuring participants completed questionnaires within their scheduled window. Clinical examiners also helped with retention activities and assisted the project manager with administrative tasks that included activities to increase participant engagement (e.g., creating “activity packets” for children), drafting new clinical procedures to comply with COVID-19 guidelines and assisting with return to research documents. All staff and clinical examiners were necessary for data collection that resumed in September 2020; this delay in timeline and associated staffing costs had significant budget impacts. Reduction in force was avoided due to the lengthy training required to replace research assistants, and that the dental hygienists were already trained and calibrated.

The PACT trial moved all staff to remote and kept them occupied with manuscript writing, checking the electronic health record for missed and upcoming visits, sending questionnaire packages home to participants who were missed at WCVs, and data entry of returned questionnaires. Because in-person data collection resumed at the end of May 2020 staffing was not affected beyond that point.

#### Other Protocol Changes

BEECON focus groups that were slotted to take place in Fall 2020 were changed to virtual focus groups that had good attendance and participation. iSmile eliminated the option of clinic data collection visits being conducted in the home. The BU IRB also required study staff to read a script about COVID-19 risks to participants.

### Domain 4: Scientific Challenges

#### Challenges With Community-Based Research Ramp-Up

At each of the participating universities, special committees were established early in the pandemic to monitor research safety during COVID-19. All studies had to submit proposals and be approved to resume research operations. These committees prioritized laboratory research. Clinical research was next. Community research was the last group to receive review and approval.

In June, 2020 UCLA approved the ramp up of research operations University wide. The BEECON study was not required to submit a Research Operational Plan to the University because the research activities took place off campus at a community health clinic. The required approval needed to come from the community health clinic, which did not approve resuming clinical visits until October 2020. UCSF created a COVID Community Advisory Board (CCAB) to review community-based research in the San Francisco Bay Area and required the CCAB approve plans to resume research; because BEECON’s performance site is in LA, the UCSF CCAB approval was not required.

In June 2020, UIC approved the CO-OP trial for restart with the same safety instructions that applied to clinical and laboratory research. The trial did not resume in-person data collection because the State of Illinois safety orders were clear that everyone should stay at home except for essential activities and the trial leadership determined that study data collection was not essential.

After iSmile submitted to the BU COVID-19 research committee for restart approval, trial leadership was informed that because the study took place in community settings, an additional proposal and protocol also needed to be submitted to the affiliated hospital’s research resumption committee. The hospital committee required that the study receive approval from each of the four study sites before granting approval.

However, each site wanted BU approval before granting approval at its site. At GSDM, laboratory and clinical research conducted within the dental school had overarching COVID-19 protocols to customize for its research. iSmile, BU GSDM’s only community-based clinical trial, had to develop its own protocols that adhered to protocols from GSDM, the hospital granting the approval and that were tailored to each site’s COVID procedures.

For PACT, the State of Ohio and CWRU deemed research as essential and allowed feasible activities to continue. Since the PACT trial was conducted using hospital-based primary care clinics, the study team had to follow hospital system guidelines. When the trial resumed, the study PI had to submit a COVID-19 plan to the CWRU leadership for approval and list study staff who would be going to the community. The hospital IRB also required a COVID-19 plan be submitted before visits could be resumed.

## DISCUSSION

The four trials in the ODHC Consortium all experienced challenges in their timelines and staffing due to COVID-19, all of which increased budget expenditures. These pediatric oral health trials were all conducted in community settings. The priority in research restart activities at all the participating institutions was on laboratory restart followed by restart of clinical research. The reasons for this are well-understood. Laboratory research includes substantial investments in animals and specimens with limited lifespans. Also, laboratory research cannot generally be conducted remotely. Clinical research often addresses life-threatening health conditions, necessitating rapid re-opening. While clinical researchers could use the COVID-19 protocols that were developed for the site where the research was operating, community research trials could not easily do the same due to the considerable variation in setting and procedures at each research site. The importance of community research was difficult for the organizations to operationalize and often organizational protocols to guide the return of community research do not exist. The other big difference is the specific risks associated with dental clinical care in the research being conducted by these four trials.

Not all changes caused by COVID-19 were negative. Some studies experienced boosts in retention and remote data collection techniques were implemented. These studies also encountered other unanticipated challenges throughout the pandemic lockdown and return to clinic which would provide helpful lessons learned to other trials navigating the COVID-19 pandemic.

The BEECON study found it difficult to navigate different levels of staff stress and anxiety about returning to clinic. Scheduling fewer participants than usual during the first 2 days at the clinic in an effort to allow the staff to get used to increased cleaning and safety protocols helped to reduce some staff anxiety. Some participants expressed that they were not comfortable returning to clinic due to fear of contracting the virus.

In particular, because many participants lacked their own transportation, they were nervous about taking public transit during the pandemic. BEECON study staff found integrating oral health into overall health was essential, and that Medical/Dental integration was an effective way for children to continue to be able to be screened for caries during routine medical visits that were allowed to occur while clinic research and preventative dental visits were not allowed to occur. The CO-OP study found that while it is feasible for parents to apply disclosing solution to their child’s teeth on their own and take adequate images, it was not easy.

The iSmile study were given fewer blocks to schedule participants at clinics. Because of the visit backlog, these blocks filled up quickly which sometimes required that participants were scheduled out-of-window.

Study staff had to be flexible when contacting participants and found that during the lockdown, participants were eager to talk with the research staff, reducing the number of follow up calls that could be made each day.

Both the Research Assistants and clinical examiners had anxiety about returning to the field, however, after the first day in the field, everyone reported feeling better about being able to return to “normal.” Return to clinic started slow with a very light participant load. This helped staff gain confidence, and acclimate to cleaning protocols and new clinic protocols. A few participants expressed concern about coming to the clinic, especially when COVID-19 cases began to rise. Some participants were reticent to schedule appointments; however, once an appointment was scheduled, it was more likely to occur than pre-COVID-19. Study staff tried to pair participants’ research appointments with their medical appointments, even when out-of-window.

The PACT study experienced several challenges related to staff being sick and having to quarantine for 2 weeks. Some parents delayed WCV visits, but there were no major changes to no-show rates.

### Guidance for Future Interruptions in Research

In case of future local/regional emergencies (e.g., blizzards, wild fires), national public health crises (e.g., PPE shortage), or global pandemics, having a plan in place to outline what a halt in research would look like and the steps needed to pivot to virtual/remote research activities would be helpful. Developing specific guidelines and return to research protocols for community research is critical. This could be achieved by having representatives experienced in community-based research involved in the process of intervention development, as some of the trials have done here (6). Having an emergency plan in place would also make IRB modifications easier. In addition, during the developmental stages of a trial, discussing emergency plans with community partners would be helpful so there is a general sense of what research activities will be allowed if the protocols had to change. While this planning would be helpful, there are many variables that could change if another public health crisis or pandemic occurred, such as type of virus, how contagious it is, and how it is transmitted. Having alternative options and scenarios for different situations that might affect trials in the future would be helpful to address these negative conditions. The implementation of telehealth and/or teledentistry in conjunction as a way to continue to be able to carry out clinical research would be a potential aid. Adding these platforms to our set of tools would be very beneficial in an effort to support ongoing and uninterrupted community-based participatory research. Ultimately, being flexible, persistent, and focused on adhering to the science and safety protocols will add consistency and provide alternative options.

Emergency plans need to consider how to best maintain treatment fidelity and internal validity of the study according to NIH guidelines during these crises (8).

## Figures and Tables

**TABLE 1 |:** Overview of challenges by domain for the 4 OHDC Consortium trials.

	BEECON	COOP	iSmile	PACT
Change in timeline	X	X	X	X
Increased retention	X		X	
Challenges to obtaining PPE		X	X	
Change to participant compensation		X	X	
Extended data collection windows	X	X	X	X
Change to data collection protocols	X	X		X
Change to handling missing data and change in analytic plan			X	

**TABLE 2 |:** Trial timelines and activities before and during COVID-19.

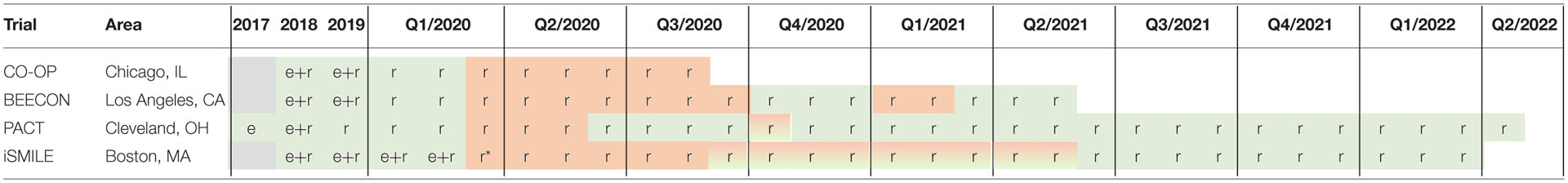

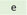
 *Enrollment/randomization*.

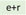
 *Enrollment/randomization and retention (follow-up visits)*.

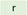
 *Retention (follow-up visits)*.

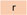
 *During COVID—remote retention (follow-up visits)*.

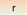
 *During COVID—partial remote retention (follow-up visits), some sites and/or some part of the month*.

* Enrollment/randomization ended March 6, 2019 (pre-COVID shutdown).

## Data Availability

The original contributions presented in the study are included in the article/supplementary material, further inquiries can be directed to the corresponding author.
